# Dual Task Performance in Normal Aging: A Comparison of Choice Reaction Time Tasks

**DOI:** 10.1371/journal.pone.0060265

**Published:** 2013-03-21

**Authors:** Eleftheria Vaportzis, Nellie Georgiou-Karistianis, Julie C. Stout

**Affiliations:** School of Psychology and Psychiatry, Faculty of Medicine, Nursing and Health Sciences, Monash University, Clayton, Victoria, Australia; University of Leicester, United Kingdom

## Abstract

This study examined dual task performance in 28 younger (18–30 years) and 28 older (>60 years) adults using two sets of choice reaction time (RT) tasks paired with digit tasks. Set one paired simple choice RT with digit forward; set two paired complex choice RT with digit backward. Each task within each set had easy and hard conditions. For the simple choice RT, participants viewed single letters and pressed a specified keyboard key if the letter was X or Z or a different key for other letters (easy). For the hard condition, there were 4 target letters (X, Z, O, Y). Digit forward consisted of 4 (easy) or 5 (hard) digits. For the complex choice RT, participants viewed 4×4 matrices of Xs and Os, and indicated whether four Xs (easy) *or* four Xs or four Os (hard) appeared in a row. Digit backward consisted of 3 (easy) or 4 (hard) digits. Within each set, participants performed every possible combination of tasks. We found that in the simple choice RT tasks older adults were significantly slower than, but as accurate as younger adults. In the complex choice RT tasks, older adults were significantly less accurate, but as fast as younger adults. For both age groups and both dual task sets, RT decreased and error rates increased with greater task difficulty. Older adults had greater dual task costs for error rates in the simple choice RT, whereas in the complex choice RT, it was the younger group that had greater dual task costs. Findings suggest that younger and older adults may adopt differential behavioral strategies depending on complexity and difficulty of dual tasks.

## Introduction

Choice reaction time (RT) tasks have been routinely used in aging research [1]–[8]. They are potent for discriminating between different age groups [1], [7], [9], are more sensitive measures of age-related differences in psychomotor performance compared with simple RT tasks [4], [10], and correlate significantly with higher-order cognitive processes in younger and older adults [8]. Previous studies have employed choice RT tasks to investigate the effect of dual tasking across various age groups [11]–[14]. The general agreement is that choice RT slows with aging and with increased task difficulty, which allegedly reflects competition for attentional resources. However, most previous aging studies have examined the effect of choice RT tasks on postural balance in healthy individuals and manipulated one of the dual tasks only [11], [13]. Our understanding of the effect of aging on cognitive task performance under different levels of dual task difficulty remains limited. Extending this area of research allows us to tease apart the various mental operations that deteriorate with increased age, and how they may be amplified by task difficulty and complexity.

There is some evidence that age-related differences in performance are minimal at lower levels of task demand; as task demand increases, performance of older adults declines relative to that of younger adults [15]. This finding is also predicted by the Compensation-Related Utilization of Neural Circuits Hypothesis [CRUNCH; 16]. According to CRUNCH, the aging brain recruits more neural resources due to processing inefficiencies in order to achieve equivalent performance to that of younger adults' brains [16], [17]. CRUNCH predicts that this compensatory neural activation is efficient at low levels of task demand, however, with higher levels of task demand, age-related differences emerge [17].

Some studies investigated age-related dual task effects under different levels of task difficulty. For example, McDowd and Craik (1988) paired cognitive choice RT tasks and manipulated task difficulty to investigate age differences in participants who were required to perform auditory and visual choice RT tasks on their own and concurrently. Both choice RT tasks had easier and harder conditions producing four dual task combinations (easy auditory choice RT with easy visual choice RT; easy auditory choice RT with hard visual choice RT; hard auditory choice RT with easy visual choice RT; hard auditory choice RT with hard visual choice RT). In a second study, using visual choice RT, complexity was further increased by manipulating the number of choices (two-, four- and eight-choice RT). Results across both studies showed that older adults had slower choice RT compared with younger adults, and their performance further deteriorated under the harder dual task conditions. There was some support for an age-related decrease in dual task costs, which amplified with increased task complexity. The authors concluded that age differences are likely to be amplified by task difficulty and complexity, perhaps because mental operations slow with increased age. This slowing is exaggerated in dual task conditions that require a greater number of operations, providing a promising avenue in which to examine the effects of increased task complexity.

The generalized slowing, observed with increased age, is predicted by the Processing-Speed Theory. This theory postulates that age-related differences, observed during cognitive task performance, are likely to be underpinned by changes in processing speed [18]. According to this theory, age-related differences should be more pronounced in more cognitively demanding tasks, which are assumed to embody several simpler cognitive processing stages. Past research has suggested that RT becomes slower and more variable with increased age; a finding supported by longitudinal studies [5], [7]. However, when measuring accuracy, the effect of age is remarkably mixed. Some studies have reported no differences in error rates between younger and older adults [19], younger adults making fewer errors [20]–[24] or more errors [25], [26]. It should be noted, however, that a limitation of several previous studies is that they either reported RT or error rates, but not both. This presents a potential limitation since including both variables offers a more holistic view of dual task performance than viewing speed and accuracy individually, allowing speed-accuracy trade-offs to be taken into account.

In light of previous findings, we sought to investigate whether age-related differences in dual task performance emerge when choice RT tasks are performed concurrently with cognitive tasks, and whether they are more pronounced with increased task complexity and difficulty. We extended past research by employing and comparing two sets of dual tasks that differed in their degree of complexity. The first task combination paired simple choice RT with digit forward (termed the *simple dual task set*), and the second combination paired complex choice RT with digit backward (termed the *complex dual task set*). We selected digit forward and backward, because both have been previously used to assess attention and working memory [27], and are comparable to each other, but vary in complexity. Previous studies have suggested that older adults perform well on short-term memory tasks that require passive storage of information (e.g., digit forward), whereas age-related impairments emerge in working memory tasks that require participants to not only hold information in memory, but also perform an operation (e.g., digit backward) [28]. Therefore, age differences are expected to be minimal in digit forward, but greater in digit backward. We also manipulated the difficulty of each task within the dual task sets, by adopting easy and hard conditions for both the choice RT and digit tasks. We predicted that older adults would be slower across all task conditions compared with younger adults, and that RT would be slower with higher levels of task difficulty across both age groups. Based on previous findings and the Processing-Speed Theory, age - related differences in RT were predicted to be more pronounced in the more complex dual task set. We also predicted that error rates would be higher with increased task difficulty across both groups.

## Methods

### Participants

Participants were 28 younger (18–30 years) and 32 older (61–90 years) adults. Four older participants were excluded due to either low scores on the Montreal Cognitive Assessment [MoCA; 29] or inability to perform some of the tasks. The final sample comprised 28 younger (15 females, *M* = 22.21, *SD*  = 3.14) and 28 older (15 females, *M* = 71.96, *SD*  = 7.84) adults. The MoCA is a 30-point cognitive screening test that emphasizes executive functioning and attention. We adopted the suggested cut-off of 26 for mild impairment. The average MoCA scores were not significantly different between the two groups (*M*Y  = 28.07, *SD*Y  = 1.58; *M*O  = 27.04, *SD*O  = 1.87), *t*(54)  = 1.79, *p* = .08.

Participants were also screened with the Wechsler Test of Adult Reading [WTAR; 30]. The WTAR consists of 50 words that have irregular letter to sound translations, and provides an estimate of verbal IQ by emphasizing previous word knowledge for correct pronunciations. There was no significant difference in the WTAR scores for the two groups (*M*Y  = 107.50, *SD*Y  = 8.55; *M*O  = 110.32, *SD*O  = 5.89), *t*(54)  = −.143, *p* = .16. The Inventory of Depressive Symptomatology-Self-report [IDS-SR; 31] was also administered to assess depression severity within the past 7 days for all criterion domains of major depression according to the Diagnostic and Statistical Manual-IV [32]. Scores can range between 0 and 84, with lower scores indicating no depressive symptoms and higher scores very severe depressive symptoms. We found no differences in the IDS-SR scores for the two groups (*M*Y  = 11.96, *SD*Y  = 7.09; *M*O  = 13.97, *SD*O  = 8.34), *t*(54)  = −.435, *p* = .67. Education level was assessed based on the International Standard Classification of Education [ISCED; 33] system (e.g., 0 =  pre-primary education; 6 =  second stage tertiary education). Younger participants had significantly more formal education than older participants, *t*(54)  = 4.81, *p*<.001; years of education was used as a covariate in all analyses.

Ethics approval was granted by the Monash University Human Research Ethics Committee. All participants gave written informed consent and reported that they had normal or corrected-to-normal vision and hearing, and that that they were free of neurological disease, psychological disorders, and upper limb impairments. They were also fluent in English, and their MoCA, WTAR and IDS-SR scores were all within the normal range.

### Dual Task Description

Participants performed two sets of dual tasks: the simple dual task set paired simple choice RT with digit forward, and the complex dual task set paired complex choice RT with digit backward. Paré, Rabin, Fogel and Pépin [34] used a similar simple choice RT task to examine dual task performance in individuals with mild traumatic brain injury. Müller et al. [35] employed a similar complex choice RT task to examine dual task performance in Huntington's disease. Each of the tasks within each set had easy *and* hard conditions. The choice RT tasks were administered on a Lenovo ThinkPad X61 laptop running Windows XP. The laptop was placed in front of the participants within comfortable reach. Participants used the laptop's keyboard to respond to stimuli that were presented in the centre of the screen: they pressed the left arrow with their left index finger to respond to target stimuli, and the right arrow with their right index finger to respond to non-target stimuli. The ratio of target to non-target stimuli was approximately the same. We recorded RT and error rates. RT was the time taken from the moment each stimulus appeared on the screen until participants' response. Error rates were the percentage of incorrect responses across trials. Responding to a target or non-target stimulus with the appropriate keyboard arrow constituted a correct response.

**Figure 1:**
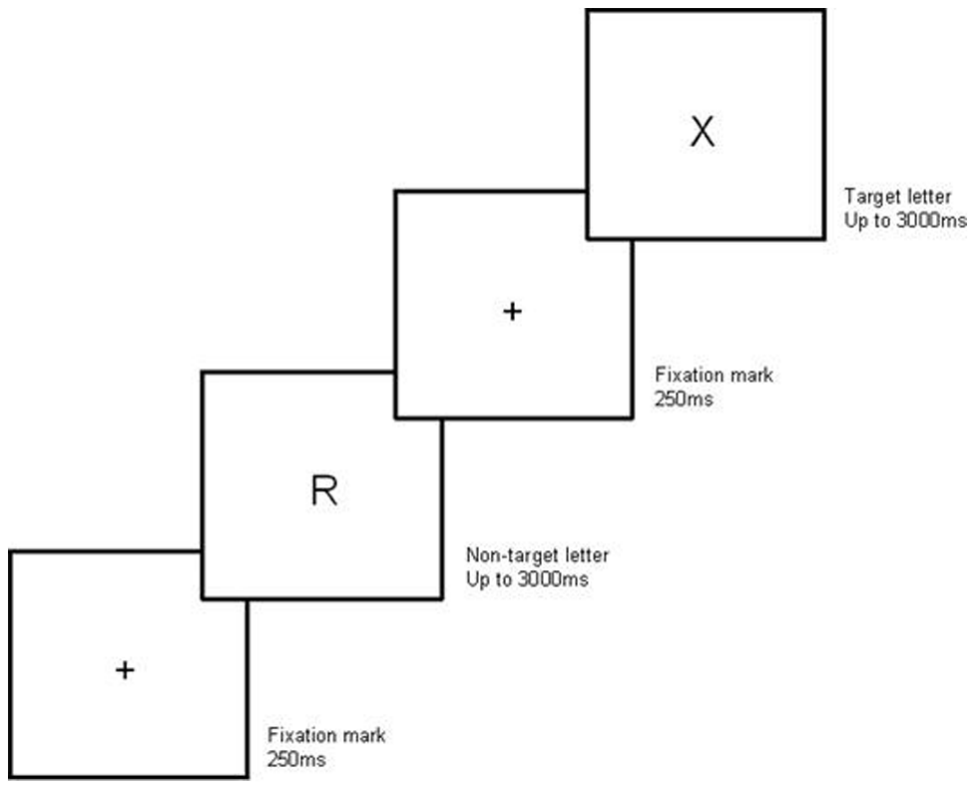
A non-target (R) and a target (X) trial of the simple choice RT task.

For *simple choice RT*, stimuli were specific letters of the alphabet, which were designated as target and non-target letters. In the easy condition the target letters were X and Z, and in the hard condition they were X, Z, O and Y. Non-target letters were other letters of the alphabet. Each trial commenced with a “get-ready” sign (+) that remained on the screen for 250 ms. A letter followed in the same position, and until the participant responded or for a maximum of 3000 ms (see Figure 1). Because hard digit forward requires more time, we adjusted the number of simple choice RT trials so that there were enough trials to last throughout the hard digit task. Thus, there were 45 simple choice RT trials performed concurrently with easy digit forward and 54 trials performed concurrently with hard digit forward.

**Figure 2:**
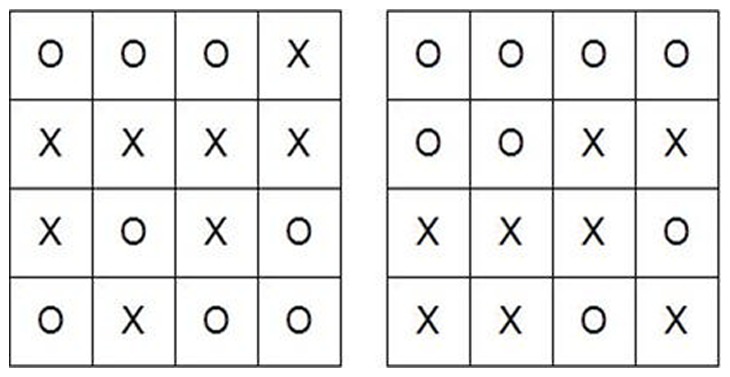
Target stimuli of the complex choice RT task conditions. On the left matrix, four Xs appear in a row (easy *or* hard conditions); on the right matrix, four Os appear in a row (hard condition).

For *complex choice RT*, stimuli were 4×4 matrices of regular arrays of eight Xs and eight Os. In the easy condition the target matrices had four Xs in a row, either horizontally, vertically, or diagonally. In the hard condition, they had either four Xs *or* four Os in a row (see Figure 2). Non-target matrices did not have four Xs or four Os in a row; they appeared in any location that did not constitute a row. Each stimulus was displayed on the screen until response was made or for a maximum of 3000 ms with an interstimulus interval of 500 ms. There were 30 complex choice RT trials when performed concurrently with easy digit backward and 40 trials performed concurrently with hard digit backward.

For *digit forward*, stimuli were 4-digit (easy) and 5-digit (hard) numbers; for *digit backward*, stimuli were 3-digit (easy) and 4-digit (hard) numbers. Digits ranged between 0 and 9, and each digit appeared only once in any given number. The experimenter read each series of numbers at a rate of approximately 1 s. Participants were then required to repeat each series aloud and in the correct order. As soon as participants recalled a series, the experimenter presented the next one. Incorrect digits or digits out of order were counted as errors. These errors were summed across trials and divided by the total number of trials in that condition to obtain the error percentage, which we then used for data analysis. There were 10 trials for the single tasks for each task set. In dual tasks, the number of trials varied from one participant to another as the digit tasks ended only when participants had completed the choice RT tasks.

For the dual task conditions, participants were required to press the Enter button on the keyboard in order to commence the choice RT tasks. As soon as they commenced each of the choice RT tasks, the experimenter started reading a series of numbers which's length depended on the condition (e.g., easy, hard). The experimenter moved on to the next series of numbers as soon as the participant recalled the previous series.

Participants were tested individually in a quiet room. They were instructed to perform all tasks as quickly and as accurately as possible. For each of the sets, participants first performed the four single tasks. Taking for example the simple dual task set, participants performed the single tasks in the following order: easy simple choice RT, hard simple choice RT, easy digit forward, and hard digit forward. Participants performed practice trials prior to each of the single tasks. Next, for the dual tasks, participants performed every possible combination of the simple choice RT with the digit forward tasks: (1) easy simple choice RT with easy digit forward, (2) easy simple choice RT with hard digit forward, (3) hard simple choice RT with easy digit forward, and (4) hard simple choice RT with hard digit forward. The same order was followed for the complex dual task set.

The simple and complex dual task sets were two of four sets of tasks that participants performed as part of a larger study, and the order of the four sets was counterbalanced across participants. Therefore, half of the participants in each of the groups performed the simple dual task set first, and the other half of participants performed the complex dual task set first. We did not counterbalance the order of the conditions, because a full permutation with all the different conditions for all the different sets of tasks was deemed impractical due to the large number of conditions within each set of tasks as well as the sample size. Also, since there is a learning component it was appropriate for the hard tasks to be preceded by easy tasks.

### Statistical Analyses

For *RT* and *error rates* across all tasks, trials with values more than 3.5 standard deviations from the individual mean were excluded before computing overall means and standard deviations (see Table 1). Taking, for example the simple dual task set, separate 2×2×3 mixed model ANOVAs were computed for RT and for errors rates, with Age as a between subjects factor (young, old), and two within factors, Simple Choice RT Task Difficulty (easy, hard), and Digit Forward Task Difficulty (none, easy, hard). The same model was used for the complex dual task set. Due to violations of the sphericity assumption, we report Greenhouse-Geisser corrected degrees of freedom. Education level was included as a covariate in all specified models below, but it was not found to be significant in any model. Thus, all subsequent analyses were performed without education as a covariate. Significant interactions of interest were followed with appropriate post hoc analyses: simple main effects or planned comparisons. For all pairwise comparisons we conducted Bonferroni post hoc tests (α = .05).

**Table 1:** Means (and standard deviations) of younger and older adults across all tasks.

	Simple Choice RT (RT)		Simple Choice RT (Errors)		Digit Forward (Errors)		Complex Choice RT (RT)		Complex Choice RT (Errors)		Digit Backward (Errors)	
	Young	Old	Young	Old	Young	Old	Young	Old	Young	Old	Young	Old
E Single Task	496	618	3.17	4.05	.00	.00	1615	1697	7.50	21.72	7.85	6.42
	(78)	(83)	(2.52)	(3.82)	(.00)	(.00)	(475)	(463)	(7.51)	(10.71)	(8.75)	(8.26)
H Single Task	518	651	3.21	2.46	5.00	5.00	2168	2235	13.33	24.16	17.14	20.71
	(73)	(87)	(2.71)	(2.36)	(7.93)	(7.45)	(644)	(633)	(7.59)	(8.63)	(16.06)	(21.59)
E Choice RT-E Digit	613	801	3.65	4.36	1.61	3.86	2425	2745	16.66	36.30	11.18	14.36
	(123)	(173)	(3.48)	(4.50)	(4.06)	(6.05)	(502)	(581)	(9.51)	(12.74)	(14.93)	(11.64)
E Choice RT-H Digit	663	801	3.84	3.66	5.86	4.57	2431	2647	22.23	35.55	13.43	12.24
	(179)	(174)	(3.76)	(2.59)	(11.50)	(7.75)	(580)	(606)	(12.36)	(10.29)	(14.84)	(15.49)
H Choice RT-E Digit	639	798	4.84	5.79	13.16	13.43	2521	2609	20.95	32.96	29.80	30.94
	(120)	(171)	(3.96)	(5.78)	(15.53)	(15.10)	(572)	(519)	(13.11)	(10.91)	(28.06)	(27.26)
H Choice RT-H Digit	695	866	6.79	7.82	14.70	14.97	2611	2755	24.10	38.88	30.77	26.67
	(194)	(211)	(3.80)	(4.56)	(16.55)	(15.83)	(432)	(398)	(14.12)	(10.86)	(29.29)	(22.71)

*Note*. RT  =  Reaction time; E =  Easy; H =  Hard.

**Figure 3:**
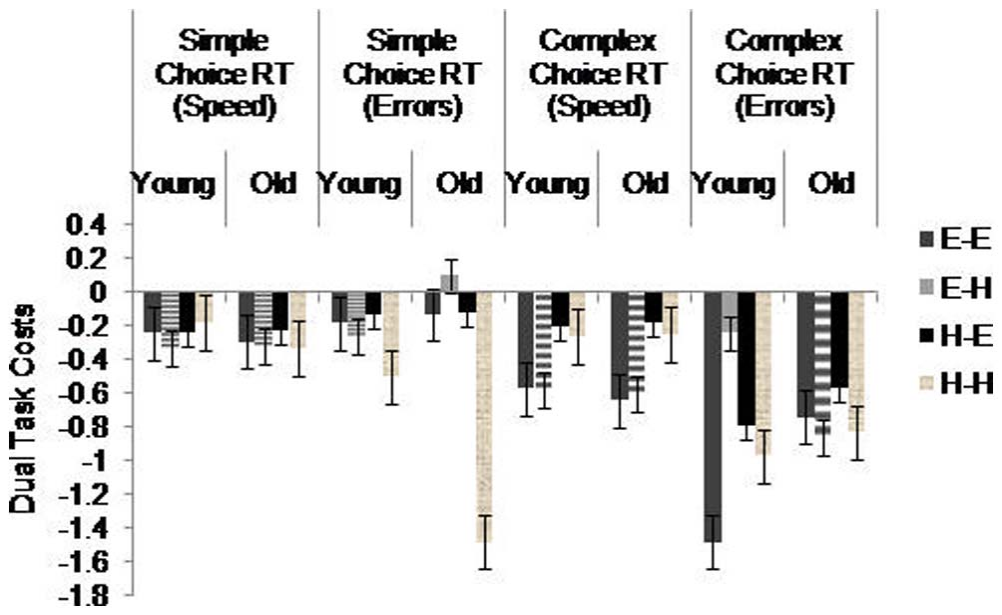
Dual task costs for the choice RT tasks. E–E  =  Easy choice RT with easy digit; E–H  =  Easy choice RT with hard digit; H–E  =  Hard choice RT with easy digit; H–H  =  Hard choice RT with hard digit. Standard error bars are included.

**Figure 4:**
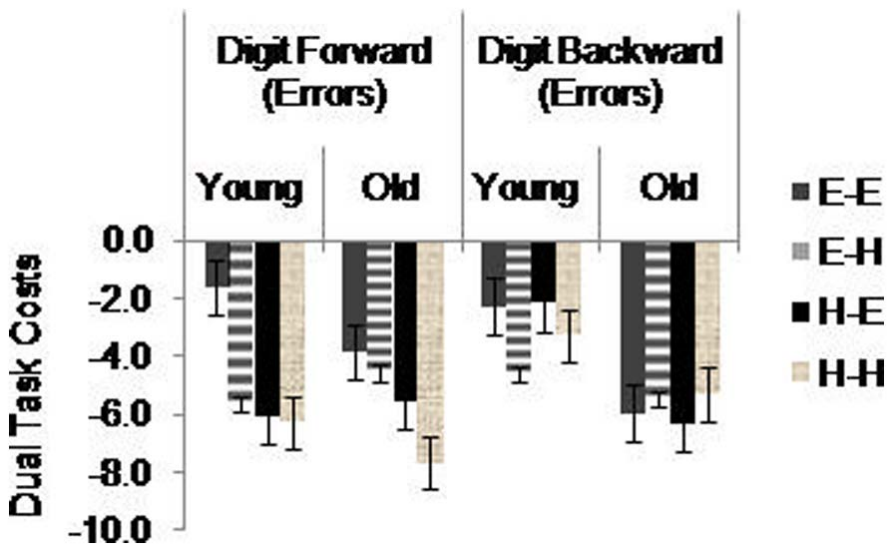
Dual task costs for the digit tasks. E–E  =  Easy digit with easy choice RT; E–H  =  Easy digit with hard choice RT; H–E  =  Hard digit with easy choice RT; H–H  =  Hard digit with hard choice RT. Standard error bars are included.

To assess participants' ability to perform concurrent tasks, we computed *dual task costs* separately for RT and error rates for the simple and complex choice RT tasks, as well as for error rates for the digit tasks (see Figures 3 and 4). Taking, for example the simple dual task set, we used a 2×2×2 mixed-model ANOVA with the between factor Age (young, old) and two within factors, Simple Choice RT Task Difficulty (easy, hard) and Digit Forward Task Difficulty (easy, hard). We used the same model for complex choice RT dual task costs. For the digit tasks, we added a value of 1 to each data point prior to computing dual task costs due to a large number of participants not committing any errors in the single tasks. In accord with previous studies [14], [36], [37] we used the formula: dual task cost  =  (single task-dual task)/single task to calculate the relative ratio of single task to dual task that controls for single task performance. Negative dual task costs suggest that RT and accuracy decreased in the dual task conditions compared with the single task conditions.

## Results

Age and condition effects on RT *and* error rates were examined in order to investigate dual task performance in younger and older adults. We first present the simple choice RT task performance followed by the complex choice RT task. Finally, we present performance on the digit tasks.

### Simple Choice RT Task Performance

**Figure 5:**
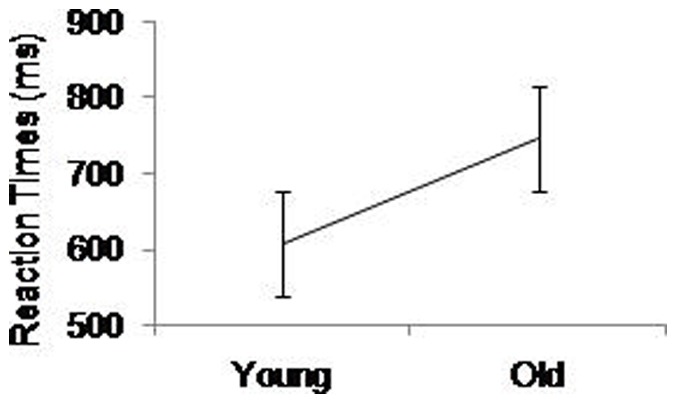
Main effect of Age (young, old) on reaction times of the simple choice RT task. Standard error bars are included.

Using RT as the dependent variable, a three way ANOVA revealed a significant main effect of Age, *F*(1,52)  = 19.55, *p*<.001, η^2^ = .27, with older adults being significantly slower than younger adults (see Figure 5). We also found significant main effects of Simple Choice RT, *F*(1.00,52.00)  = 9.88, *p* = .01, η^2^ = .16, with significantly faster performance in easy compared with hard simple choice RT, and Digit Forward, *F*(1.39,72.57)  = 72.09, *p*<.001, η^2^ = .58, with significantly faster performance in the single digit tasks compared with the dual digit tasks. Easy digit forward conditions were also performed significantly (*p* = .03) faster than hard digit forward conditions. There were no significant interactions.

**Figure 6:**
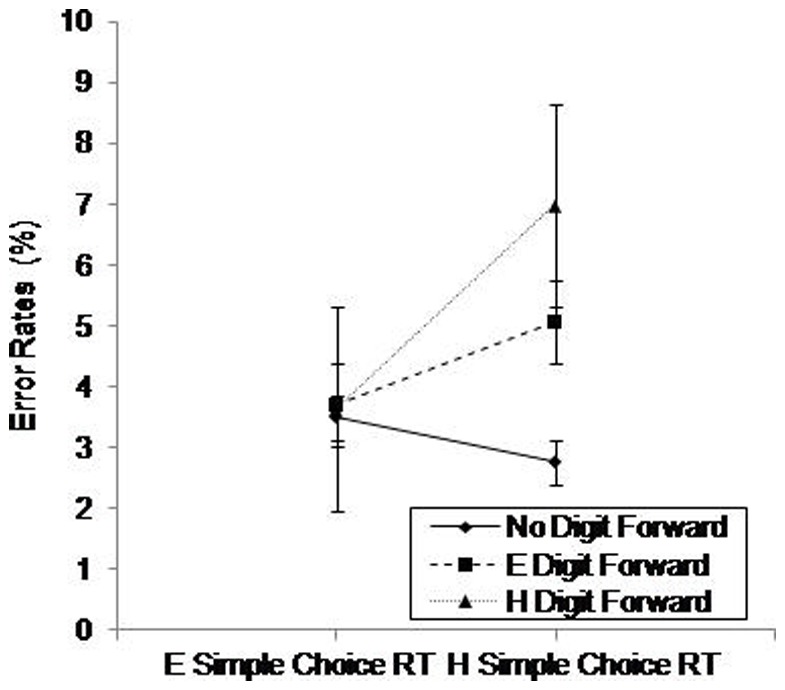
Percentage of error rates on the Simple Choice RT (easy, hard) as a function of Digit Forward Task Difficulty (none, easy, hard). E =  Easy; H =  Hard. Standard error bars are included.

Using error rates as the dependent variable, the same model revealed significant main effects of Simple Choice RT, *F*(1.00,50.00)  = 15.63, *p*<.001, η^2^ = .23 and Digit Forward, *F*(1.88,94.03)  = 11.54, *p*<.001, η^2^ = .18. There was also a significant interaction between Simple Choice RT and Digit Forward, *F*(1.90,95.31)  = 12.89, *p*<.001, η^2^ = .20 (see Figure 6). Post hoc analysis of the simple main effects showed no differences on the easy simple choice RT. For hard simple choice RT we found significant (*p*<.05) differences between the single and dual tasks, and between the easy and hard digit forward conditions; error rates increased with increased difficulty. Overall, the results of the simple choice RT tasks suggest aging effects in RT, but not in error rates. There was no evidence of speed-accuracy trade-offs, as slower responses were associated with greater error rates.

### Simple Choice RT Dual Task Costs

**Figure 7:**
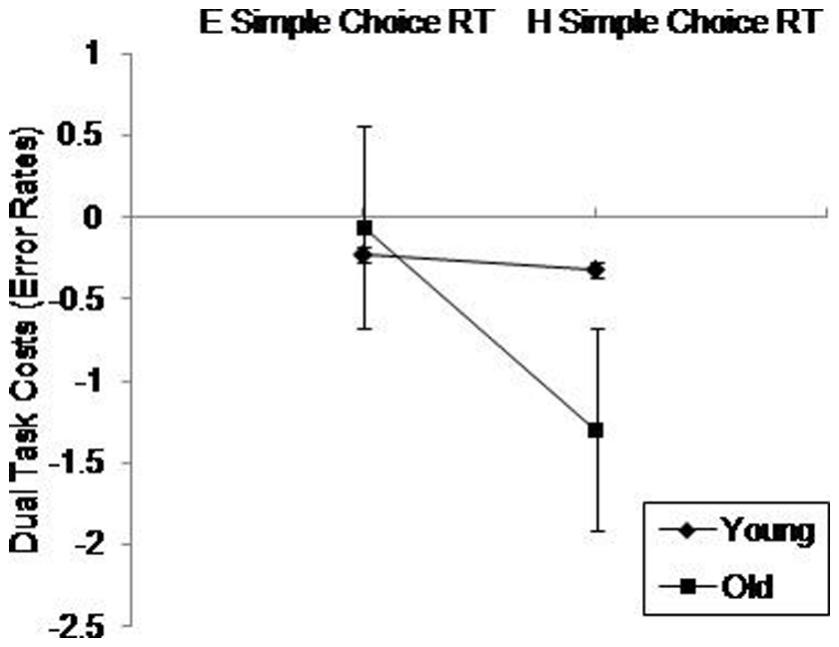
Dual task costs (error rates) on the Simple Choice RT Task Difficulty (easy, hard) as a function of Age (young, old). E =  Easy; H =  Hard. Standard error bars are included.

For dual task costs, a three way ANOVA for RT revealed a significant main effect of Digit Forward, *F*(1.00,54.00)  = 7.58, *p* = .01, η^2^ = .12, with significantly greater dual task costs in easy digit forward compared with hard digit forward. The same model for error rates revealed a significant main effect of Simple Choice RT, *F*(1.00,54.00)  = 9.86, *p* = .01, η^2^ = .15, and a significant interaction between Age and Simple Choice RT, *F*(1.00,54.00)  = 7.31, *p* = .01, η^2^ = .12 (see Figure 7). As indicated by pairwise comparisons, older adults had significantly (*p*<.001) more costs in the hard simple choice RT, whilst the other conditions had similar costs. We found no age difference on easy simple choice RT.

### Complex Choice RT Task Performance

**Figure 8:**
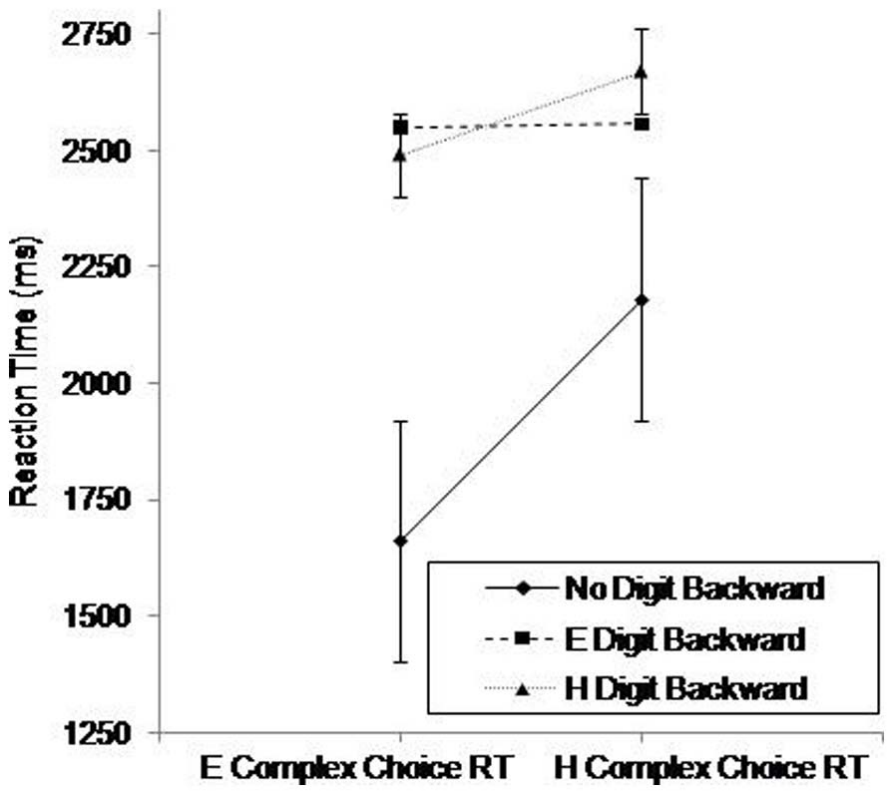
Reaction times on the Complex Choice RT (easy, hard) as a function of Digit Backward Task Difficulty (none, easy hard). E =  Easy; H =  Hard. Standard error bars are included.

Using RT as the dependent variable, a three way ANOVA revealed significant main effects of Complex Choice RT, *F*(1.00,52.00)  = 39.59, *p*<.001, η^2^ = .43, and Digit Backward, *F*(1.31,68.58)  = 112.27, *p*<.001, η^2^ = .68, and a significant interaction between Complex Choice RT and Digit Backward (see Figure 8). Post hoc analysis of the simple main effects showed that easy complex choice RT was performed significantly (*p*<.001) faster than hard complex choice RT only in the single tasks.

**Figure 9:**
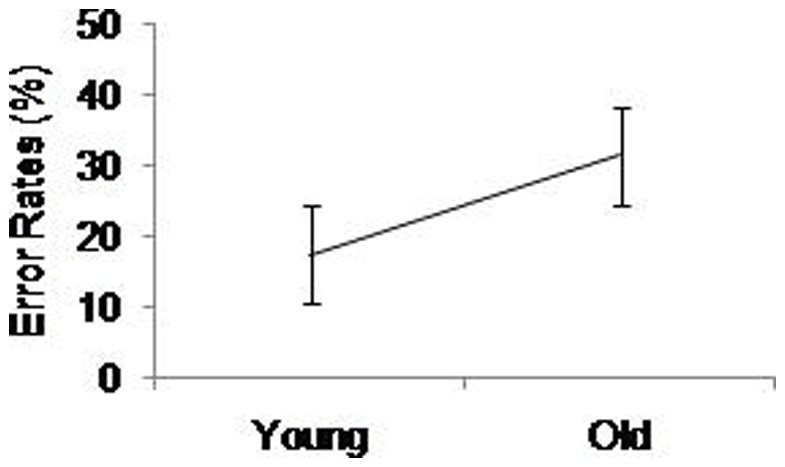
Main effect of Age (young, old) on error rates of the complex choice RT task. Standard error bars are included.

Using error rates as the dependent variable, the same model revealed a significant main effect of Age, *F*(1,50)  = 47.85, *p*<.001, η^2^ = .48, with older adults making significantly more errors than younger adults (see Figure 9). We also found significant main effects of Complex Choice RT, *F*(1.00,52.00)  = 11.21, *p* = .002, η^2^ = .17, with significantly more errors in the easy than hard complex choice RT conditions; and Digit Backward, *F*(1.47,76.61)  = 11.54, *p*<.001, η^2^ = .48. Participants made significantly fewer errors in the single tasks compared with the dual tasks, and also significantly fewer errors in easy compared with hard digit backward. Contrary with the simple choice RT tasks, the results of the complex choice RT tasks suggest aging effects in error rates, but not in RT. Similarly with the simple choice RT task, slower responses were associated with greater error rates suggesting no speed-accuracy trade-offs.

### Complex Choice RT Dual Task Costs

**Figure 10:**
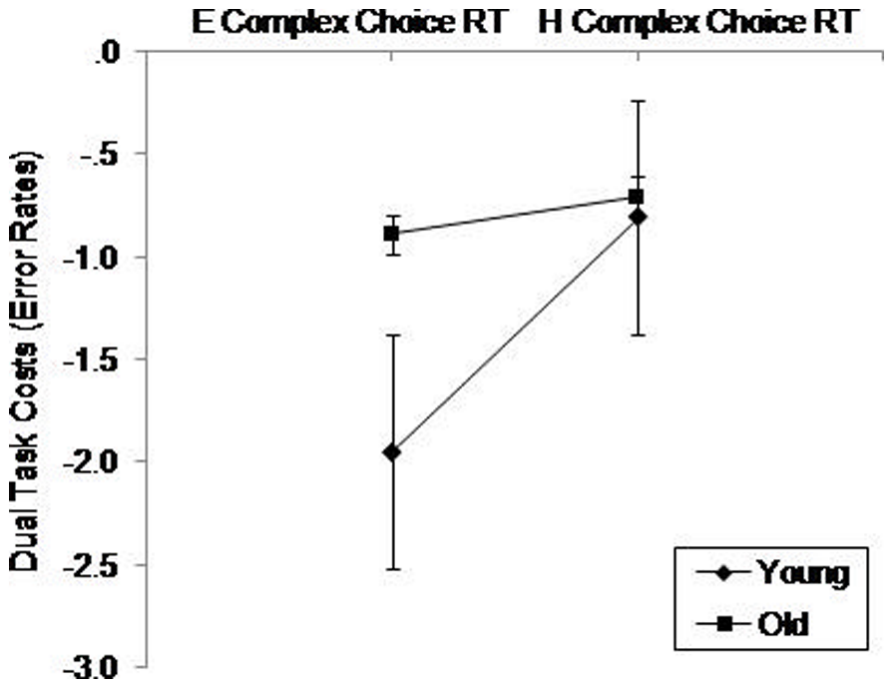
Dual task costs (error rates) on the Complex Choice RT Task Difficulty (easy, hard) as a function of Age (young, old). E =  Easy; H =  Hard. Standard error bars are included.

For dual task costs, a three way ANOVA for RT revealed only a significant main effect of Complex Choice RT, *F*(1.00,54.00)  = 33.81, *p*<.001, η^2^ = .382. The same model for error rates revealed significant main effects of Complex Choice RT, *F*(1.00,54.00)  = 6.44, *p* = .01, η^2^ = .11, and Digit Backward, *F*(1.00,54.00)  = 17.36, *p*<.001, η^2^ = .24. There was also a significant interaction between Age and Complex Choice RT, *F*(1.00,54.00)  = 4.31, *p* = .04, η^2^ = .07 (see Figure 10). Pairwise comparisons suggested that there were significantly (*p*<.001) greater dual task costs for the younger adults in the easy complex choice RT tasks.

In order to see whether there were differences in dual task costs between the simple and complex choice RT tasks, we calculated dual task cost ratios for RT and error rates separately for each condition using the following formula: simple choice RT/complex choice RT. We found that the majority of both younger and older participants had greater costs in the complex choice RT, with only very few participants having similar costs between the two tasks or greater costs on the simple choice RT. Mann-Whitney tests showed no significant age-related differences between dual task cost ratios for any of the conditions.

### Digit Tasks Performance

For *digit forward*, a three way ANOVA revealed a significant main effect of Digit Forward, *F*(1.00,49.00)  = 37.49, *p*<.001, η^2^ = .43, with significantly fewer errors in the easy than hard conditions. There was also a significant main effect of Simple Choice RT, *F*(1.82,75.71)  = 17.47, *p*<.001, η^2^ = .26, with significantly fewer errors in the single tasks compared with the dual tasks. There were no significant interactions.

For *digit backward*, the same model revealed a significant main effect of Digit Backward, *F*(1.00,52.00)  = 53.62, *p*<.001, η^2^ = .51, with significantly fewer errors in easy than hard conditions. We also found a significant main effect of Complex Choice RT, *F*(1.54,80.43)  = 13.39, *p*<.001, η^2^ = .20, with significantly fewer errors in the single tasks compared with the dual tasks. There were no significant interactions.

For dual task costs of digit forward, we found a significant main effect of Digit Forward, *F*(1.00,54.00)  = 5.18, *p* = .02, η^2^ = .08, with significantly greater dual task costs in hard digit forward compared with easy digit forward. We found no other significant main effects or interactions for digit forward or digit backward.

## Discussion

This study examined whether concurrent performance of choice RT and cognitive tasks varies depending on differences in task complexity and difficulty in younger and older adults. We found age-related differences in speed, but not in accuracy in the simple choice RT tasks with older adults having greater dual task costs only in the hard simple choice RT conditions. For the complex choice RT tasks, we found age-related differences in accuracy, but not in speed with younger adults having greater dual task costs only in the easy complex choice RT conditions. Collectively, our results suggest that younger and older adults differ in their dual task performance, with differences depending on both the complexity and difficulty of dual tasks probably due to implementation of different strategies.

Most previous studies, using different combinations of dual tasks, found that older adults were slower than younger adults [7], [13]. Our findings indicate that under more cognitively demanding conditions, age differences in speed may be eliminated, albeit at the expense of accuracy. Despite our instructions, which put equal emphasis on the importance of both speed and accuracy, older adults may have either opted for a more careful approach or could not perform the choice RT tasks any faster due to generalized slowing as suggested by Processing-Speed Theory [18]. However, age differences in speed were not more pronounced in the complex dual task set that was presumably more cognitively demanding, a finding not in support of the Processing-Speed Theory [18], although age differences in accuracy emerged. It may be possible that under the more cognitively demanding conditions younger adults slowed down due to the increased attentional demands of the tasks in order to attain a level of satisfactory accuracy. Overall, the results indicate age-differences in the strategy adopted by different groups. In support of this notion, Davidson, Amso, Cruess Anderson and Diamond [38] have also found that younger adults adjusted their speed to preserve reasonable accuracy on difficult trials.

In any case, we found no age differences in dual task costs for speed in either the simple or the complex dual task sets. Thus, both groups maintained their baseline levels of speed during the dual task conditions. In line with previous studies [14], [39], the accuracy of older adults for dual task costs decreased from baseline to the harder simple choice RT dual tasks compared with younger adults. However, a different pattern emerged in the complex choice RT tasks during which accuracy of younger adults decreased from baseline to the harder dual tasks compared with older adults, although in the context of the results in speed. This finding is unexpected given that past research has typically found that older adults incur greater dual task costs [39]. Despite this, there are a few studies that have found greater dual task costs in younger adults [e.g., 40, 41]. For example, Kemper et al. (2003) investigated language production and suggested that younger adults may experience greater dual task costs than older adults due to their faster and more complex speech at baseline, which became similar to older adults' speech under the more demanding dual task conditions. Similarly, our findings suggest that performing the hard choice RT concurrently with a secondary task had more detrimental effects on the performance of younger than older adults. This is most likely due to their comparatively lower error rates at baseline that increased under dual task conditions. Taken as a whole, our findings indicate that dual task costs are affected by the complexity of the task being performed. Although our participants were able to maintain their baseline RT under dual task conditions, their accuracy changed. One might assume that increasing task complexity changed participants' response from emphasizing speed to emphasizing accuracy; however, this is unlikely, as we found no speed-accuracy trade-offs. Therefore, our participants' accuracy decreased under more demanding dual task conditions, and baseline speed was maintained.

Previous neuroimaging studies have investigated the neural basis of processing speed [42], [43], as well as patterns of cortical activation [44], [45]. Recently, Takeuchi et al. [46] used functional magnetic resonance imaging to examine differences between the speed of simple and complex working memory tasks in 23 healthy males using an N-Back task. Significantly increased activation of the right dorsolateral prefrontal cortex and fronto-parietal network was found during faster and more complex tasks, compared with the slower and easier tasks. The authors suggested that these regions may mediate differences between the speed of simple and complex cognitive processes in line with other studies that have found increased cognitive load to be associated with increased brain activation specifically in the prefrontal cortex [47]–[49]. Moreover, past research has shown a positive relationship between speed of complex cognitive processes and psychometric measures of intelligence [e.g., 50]. The fronto-parietal network has been typically ascribed to cognitive functions, and has been found to be over-activated in older compared with younger adults [51], [52], and during dual-task performance [52], [53]. Age-related increased activation may reflect a compensatory strategy employed by older adults as an attempt to maintain task performance at an accurate level, especially under more cognitively demanding conditions. Overall, these studies emphasize the importance of the fronto-parietal network as playing a critical role in age-related differences in the speed of both simple and complex cognitive processes.

Consistent with our hypothesis, and past research [11], [13], our findings showed that performance slowed and became less accurate with higher levels of task difficulty in both groups and across both sets of tasks. Both groups were slower and made more errors in the choice RT tasks when they performed the digit tasks concurrently, highlighting the increased cognitive load in the dual task conditions. Past research investigating age differences in dual tasking has produced inconsistent results in regards to error rates [19], [21], [24]. Our findings suggest that age-related differences in error rates emerge under more complex dual tasks. Older adults' already slowed RT may have provided a “protective” mechanism in the more complex dual task set (against the need to slow down even further); however, maintaining RT was at the expense of accuracy. Alternatively, older adults may not be able to regulate their RT as effectively as younger adults to adapt to different conditions. Although results suggested no speed-accuracy trade-offs within each set of tasks, there were age-related differences in RT, but not in error rates in the simple set of tasks, and age-related differences in error rates, but not in RT in the complex set of tasks. Differences in the pattern of age effects across sets of dual tasks suggest that different age groups may implement differential strategies depending on the type and complexity of tasks.

Our study has implications for the Processing-Speed Theory. For example, Salthouse [54] argues that age-related differences in cognitive performance, such as working memory, can be explained by age-related differences in processing speed. Our results indicate that age-related differences are not explained solely by processing speed. Rather, we find clear age-related differences that are best demonstrated by two measures, RT and accuracy, with the relationships between these measures and aging varying with levels of task difficulty. Under conditions of simple processing, older participants' RT was slower relative to younger participants, whereas accuracy was similar; however, under harder processing conditions older participants' RT was similar to younger participants, but they were less accurate. These findings are consistent with past research which asserts that participants are likely to share resources between a number of factors, including processing speed and processing accuracy [55].

The current findings showed that older adults performed worse overall in both simple and complex choice RT tasks, but employed different strategies with speed-accuracy trade-offs based on the complexity of dual tasks: older adults traded speed for accuracy in the simple dual task set, and accuracy for speed in the complex dual task set. Accumulator models of speed-accuracy trade-offs assume that sensory evidence accumulates over time from signal onset until a decision threshold [56]. Depending on the task and individual differences, such as capability and age, accumulation of evidence may proceed more or less slowly and more or less accurately. Relevant to our results, accumulator models predict either changes in speed or accuracy by changing the decision threshold: emphasizing the speed of responding lowers the decision threshold relative to emphasizing accuracy and vice versa [56]. Overall, our results highlight the need to characterise dual task performance using a more comprehensive readout of behaviour, including speed and accuracy, and to include conditions that span a variety of difficulty levels. It remains to be determined whether the different pattern of results across the simple and complex choice RT tasks is under conscious control.

Our findings should be considered in light of some study limitations. Despite instructing participants to perform the tasks as quickly and as accurately as possible, it is likely that the complexity of tasks affected the competing goals of speed and accuracy differently. In addition, our conclusions must be tempered by possible task order effects since, for practical reasons, we did not counterbalance single and dual task conditions. However, given that practice effects would be expected to accrue with increased experience, together with the fact that participants' performances actually deteriorated in the higher task difficulty conditions which were presented later (i.e., with the greatest amount of practice), suggests that, if anything, counterbalancing would have strengthened our findings.

An important contribution of this study was to compare different combinations of cognitive dual tasks, and to manipulate the task difficulty of both tasks within each set, so as to further tease apart mental operations in younger and older adults. Most previous studies have used one dual task only, and manipulated task difficulty of only one of the tasks. In addition, previous studies have typically employed choice RT tasks concurrently with postural balance tasks rather than cognitive tasks.

In summary, our findings suggest that under dual task demands, older adults adopt different strategies than younger adults, and these depend on both complexity and difficulty level of the cognitive tasks. The differential pattern of performance across the lifespan affects both processing speed and processing accuracy. Compared with younger adults, older adults were significantly slower than, but as accurate in simple choice RT tasks, and significantly less accurate, but as fast in complex choice RT tasks. RT decreased and error rates increased with greater task difficulty for both age groups, and both dual task sets. Finally, older adults showed greater costs for error rates in the simple choice RT tasks, whereas in the complex choice RT tasks, it was the younger group that showed greater costs. Findings suggest that younger and older adults may adopt differential behavioral strategies depending on complexity and difficulty of dual tasks.

## References

[1] Der G, Deary IJ (2006) *Age and sex differences in reaction time in adulthood: Results from the United Kingdom Health and Lifestyle Survey* . Psychology and Aging 21: 62–73. 16594792 10.1037/0882-7974.21.1.62

[2] Williams BR, Hultsch DF, Strauss EH, Hunter MA, Tannock R (2005) *Inconsistency in reaction time across the life span* . Neuropsychology 19: 88–96. 15656766 10.1037/0894-4105.19.1.88

[3] Kolev V, Falkenstein M, Yordanova J (2006) *Motor-response generation as a source of aging-related behavioural slowing in choice-reaction tasks* . Neurobiology of Aging 27: 1719–1730. 16246465 10.1016/j.neurobiolaging.2005.09.027

[4] Yordanova J, Kolev V, Hohnsbein J, Falkenstein M (2004) *Sensorimotor slowing with ageing is mediated by a functional dysregulation of motor-generation processes: Evidence from high-resolution event-related potentials* . Brain 127: 351–362. 14607784 10.1093/brain/awh042

[5] Deary IJ, Der G (2005) *Reaction time, age, and cognitive ability: Longitudinal findings from age 16 to 63 years in representative population samples* . Aging, Neuropsychology, and Cognition 12: 187–215.

[6] Deary IJ, Johnson W, Starr JM (2010) *Are processing speed tasks biomarkers of cognitive aging?* . Psychology and Aging 25: 219–228. 20230141 10.1037/a0017750

[7] Deary IJ, Der G (2005) *Reaction time, age, and cognitive ability: Longitudinal findings from age 16 to 63 years in representative population samples* . Aging, Neuropsychology, and Cognition 12: 187–215.

[8] Deary IJ, Der G, Ford G (2001) *Reaction times and intelligence differences: A population-based cohort study* . Intelligence 29: 389–399.

[9] Moore H, Dudchenko P, Bruno JP, Sarter M (1992) *Toward modeling age-related changes of attentional abilities in rats: Simple and choice reaction time tasks and vigilance* . Neurobiology of Aging 13: 759–772. 1362798 10.1016/0197-4580(92)90100-c

[10] Benton A (1986) *Reaction time in brain disease: Some reflections* . Cortex 22: 129–140. 3709189 10.1016/s0010-9452(86)80037-5

[11] Shumway-Cook A, Woollacott M (2000) *Attentional demands and postural control: The effect of sensory context* . The Journals of Gerontology 55A: M10–M16. 10.1093/gerona/55.1.m10 10719767

[12] Mendelson DN, Redfern MS, Nebes RD, Jennings JR (2010) *Inhibitory processes relate differently to balance/reaction time dual tasks in young and older adults* . Aging, Neuropsychology, and Cognition 17: 1–18. 10.1080/13825580902914040 PMC4904295 19526388

[13] Huxhold O, Li S-C, Schmiedek F, Lindenberger U (2006) *Dual-tasking postural control: Aging and the effects of cognitive demand in conjunction with focus of attention* . Brain Research Bulletin 69: 294–305. 16564425 10.1016/j.brainresbull.2006.01.002

[14] McDowd JM, Craik FIM (1988) *Effects of aging and task difficulty on divided attention performance* . Journal of Experimental Psychology: Human Perception and Performance 14: 267–280. 2967880 10.1037/0096-1523.14.2.267

[15] Mattay VS, Fera F, Tessitore A, Hariri AR, Das S, et al (2002) *Neurophysiological correlates of age-related changes in human motor function* . Neurology 58: 630–635. 11865144 10.1212/wnl.58.4.630

[16] Reuter-Lorenz PA, Lustig C (2005) *Brain aging: Reorganizing discoveries about the aging mind* . Current Opinion in Neurobiology 15: 245–251. 15831410 10.1016/j.conb.2005.03.016

[17] Reuter-Lorenz PA, Cappell KA (2008) *Neurocognitive aging and the compensation hypothesis* . Current Directions in Psychological Science 17: 177–182.

[18] Salthouse TA (1996) The *processing-speed theory of adult age differences in cognition* . Psychological Review 103: 403–428. 8759042 10.1037/0033-295x.103.3.403

[19] Hawkins HL, Kramer AF, Capaldi D (1992) *Aging, exercise, and attention* . Psychology and Aging 7: 643–653. 1466833 10.1037//0882-7974.7.4.643

[20] Springer S, Giladi N, Peretz C, Yogev G, Simon ES, et al (2006) *Dual-tasking effects on gait variability: The role of aging, falls, and executive function* . Movement Disorders 21: 950–957. 16541455 10.1002/mds.20848

[21] Hein G, Schubert T (2004) *Aging and input processing in dual-task situations* . Psychology and Aging 19: 416–432. 15382993 10.1037/0882-7974.19.3.416

[22] McPhee LC, Scialfa CT, Dennis WM, Ho G, Caird JK (2004) *Age differences in visual search for traffic signs during a simulated conversation* . Human Factors 46: 674–685. 15709329 10.1518/hfes.46.4.674.56817

[23] Mutter SA, Goedert KM (1997) *Frequency discrimination vs frequency estimation: Adult age differences and the effect of divided attention* . The Journal of Gerontology 52B: P319–P328. 10.1093/geronb/52b.6.p319 9403521

[24] Cho C-Y, Gilchrist L, White S (2008) *A comparison between young and old adults in their ability to rapidly sidestep during gait when attention is divided* . Gerontology 54: 120–127. 18303239 10.1159/000118603

[25] Bherer L, Kramer AF, Peterson MS, Colcombe S, Erickson K, et al (2008) *Transfer effects in task-set cost and dual-task cost after dual-task training in older and younger adults: Further evidence for cognitive plasticity in attentional control in late adulthood* . Experimental Aging Research 34: 188–219. 18568979 10.1080/03610730802070068 PMC2845439

[26] Kemper S, Schmalzried R, Herman R, Leedahl S, Mohankumar D (2009) *The effects of aging and dual task demands on language production* . Aging, Neuropsychology, and Cognition 16: 241–259. 10.1080/13825580802438868 PMC2674132 18982506

[27] Grégoire J, Van Der Linden M (1997) *Effect of age on forward and backward digits* . Aging, Neuropsychology, and Cognition 4: 140–149.

[28] Babcock RL, Salthouse TA (1990) *Effects of increased processing demands on age differences in working memory* . Psychology and Aging 5: 421–428. 2242246 10.1037//0882-7974.5.3.421

[29] Nasreddine ZS, Phillips NA, Bédiriam V, Charbonneau S, Whitehead V, et al (2005) *The Montreal Cognitive Assessment, MoCA: A brief screening tool for mild cognitive impairment* . Journal of the American Geriatrics Society 53: 695–699. 15817019 10.1111/j.1532-5415.2005.53221.x

[30] Wechsler D (2001) *Wechsler Test of Adult Reading*. San Antonio: The Psychological Corporation.

[31] Rush AJ, Carmody T, Reimitz P-E (2006) *The Inventory of Depressive Symptomatology (IDS): Clinician (IDS-C) and Self-Report (IDS-SR) ratings of depressive symptoms* . International Journal of Methods in Psychiatric Research 9: 45–59.

[32] American Psychiatric Association (1994) *Diagnostic and statistical manual of mental disorders* Fourth edition Washington DC: American Psychiatry Association.

[33] UNESCO (1997) *International Standard Classification of Education* Available from: http://www.uis.unesco.org/ev.php?ID=7433_201&ID2=DO_TOPIC.

[34] Paré N, Rabin LA, Fogel J, Pépin M (2009) *Mild traumatic brain injury and its sequelae: Characterisation of divided attention deficits* . Neuropsychological rehabilitation 19: 110–137. 18609010 10.1080/09602010802106486

[35] Müller SV, Jung A, Preinfalk J, Kolbe H, Ridao-Alonso M, et al (2002) *Disturbance of “extrinsic alertness” in Huntingtons disease* . Journal of Clinical and Experimental Neuropsychology 24: 517–526. 12187464 10.1076/jcen.24.4.517.1043

[36] Swanenburg J, de Bruin ED, Hegemann S, Uebelhart D, Mulder T (2010) *Dual tasking under compromised visual and somatosensory input in elderly fallers and non-fallers* . The Open Rehabilitation Journal 3: 169–176.

[37] de Ribaupierre A, Ludwig C (2003) *Age differences and divided attention: Is there a general deficit?* . Experimental Aging Research 29: 79–105. 12735083 10.1080/03610730303705

[38] Davidson MC, Amso D, Cruess Anderson L, Diamond A (2003) *Development of cognitive control and executive functions from 4 to 13 years: Evidence from manipulations of memory, inhibition, and task switching* . Neuropsychologia 44: 2037–2078. 10.1016/j.neuropsychologia.2006.02.006 PMC1513793 16580701

[39] Loose R, Kaufmann C, Auer DP, Lange KW (2003) *Human prefrontal and sensory cortical activity during divided attention tasks* . Human Brain Mapping 18: 249–259. 12632463 10.1002/hbm.10082 PMC6871829

[40] Kemper S, Hoffman L, Schmalzried R, Herman R, Kieweg D (2011) *Tracking talking: Dual task costs of planning and producing speech for young versus older adults* . Aging, Neuropsychology, and Cognition 18: 257–279. 10.1080/13825585.2010.527317 PMC3091967 21140310

[41] Matthews A, Garry MI, Martin F, Summers J (2006) *Neural correlates of performance trade-offs and dual-task interference in bimanual coordination: An ERP investigation* . Neuroscience Letters 400: 172–176. 16530954 10.1016/j.neulet.2006.02.043

[42] Menon V, Rivera SM, White CD, Glover GH, Reiss AL (2000) *Dissociating prefrontal and parietal cortex activation during arithmetic processing* . Neuroimage 12: 357–365. 10988030 10.1006/nimg.2000.0613

[43] Loose R, Kaufmann C, Tucha O, Auer DP, Lange KW (2006) *Neural networks of response shifting: Influence of task speed and stimulus material* . Brain Research 1090: 146–155. 16643867 10.1016/j.brainres.2006.03.039

[44] Wolf RC, Vasic N, Schönfeldt-Lecuona C, Landwehrmeyer B, Ecker D (2007) *Dorsolateral prefrontal cortex dysfunction in presymptomatic Huntington's disease: Evidence from event-related fMRI* . Brain 130: 2845–2857. 17855375 10.1093/brain/awm210

[45] Barch DM, Braver TS, Cohen JD, Nystrom LE, Forman SD, et al (1997) *Dissociating working memory from task difficulty in human prefrontal cortex* . Neuropsychologia 35: 1373–1380. 9347483 10.1016/s0028-3932(97)00072-9

[46] Cohen JD, Perlstein WM, Braver TS, Casey BJ, Servan-Schreiber D, et al (1997) *Temporal dynamics of brain activation during a working memory task* . Nature 386: 604–608. 9121583 10.1038/386604a0

[47] Bors DA, Forrin B (1995) *Age, speed of information processing, recall, and fluid intelligence* . Intelligence 20: 229–248.

[48] Van Impe A, Coxon JP, Goble DJ, Wenderoth N, Swinnen SP (2011) *Age-related changes in brain activation underlying single- and dual-task performance: Visuomanual drawing and mental arithmetic* . Neuropsychologia 49: 2400–2409. 21536055 10.1016/j.neuropsychologia.2011.04.016

[49] Goble DJ, Coxon JP, Van Impe A, De Vos J, Wenderoth N, et al (2010) *The neural control of bimanual movements in the elderly: Brain regions exhibiting age-related increases in activity, frequency-induced neural modulation, and task-specific compensatory recruitment* . Human Brain Mapping 31: 1281–1295. 20082331 10.1002/hbm.20943 PMC6871108

[50] D' Esposito M, Detre JA, Alsop DC, Shin RK, Atlas S, et al (1995) *The neural basis of the central executive system of working memory* . Nature 378: 279–281. 7477346 10.1038/378279a0

[51] Verhaeghen P, Steitz DW, Sliwinski MJ, Cerella J (2003) *Aging and dual-task performance: A meta-analysis.* . Psychology and Aging 18: 443–460. 14518807 10.1037/0882-7974.18.3.443

[52] Kemper S, Herman RE, Lian CHT (2003) *The costs of doing two things at once for young and older adults: Talking while walking, finger tapping, and ignoring speech or noise* . Psychology and Aging 18: 181–192. 12825768 10.1037/0882-7974.18.2.181

[53] Murray LL, Holland AL, Beeson PM (1998) *Spoken language of individuals with mild fluent aphasia under focused and divided-attention conditions* . Journal of Speech, Language & Hearing Research 41: 213–227. 10.1044/jslhr.4101.213 9493746

[54] Salthouse TA (1994) *The aging of working memory.* . Neuropsychology 8: 535–543.

[55] Brébion G (2003) *Working memory, language comprehension, and aging: Four experiments to understand the deficit* . Experimental Aging Research 29: 269–301. 12775439 10.1080/03610730303725

[56] Ivanoff J, Branning P, Marois R (2008) *fMRI evidence for a dual process account of the speed-accuracy tradeoff in decision-making* . PLoS ONE 3: e2635. 18612380 10.1371/journal.pone.0002635 PMC2440815

